# Clinical indicators to identify neuropathic pain in low back related leg pain: a modified Delphi study

**DOI:** 10.1186/s12891-020-03600-y

**Published:** 2020-09-08

**Authors:** Jai Mistry, Deborah Falla, Tim Noblet, Nicola R. Heneghan, Alison Rushton

**Affiliations:** 1 St Georges Hospital NHS Foundation Trust, London, UK; 2 Centre of Precision Rehabilitation for Spinal Pain (CPR Spine), School of Sport, Exercise and Rehabilitation Sciences, College of Life and Environmental Sciences, University of Birmingham, Birmingham, UK

**Keywords:** Neuropathic pain, Low back related leg pain, Clinical indicators, Delphi

## Abstract

**Background:**

Neuropathic pain (NP) is common in patients presenting with low back related leg pain. Accurate diagnosis of NP is fundamental to ensure appropriate intervention. In the absence of a clear gold standard, expert opinion provides a useful methodology to progress research and clinical practice. The aim of this study was to achieve expert consensus on a list of clinical indicators to identify NP in low back related leg pain.

**Methods:**

A modified Delphi method consisting of three rounds was designed in accordance with the Conducting and Reporting Delphi Studies recommendations. Recruitment involved contacting experts directly and through expressions of interest on social media. Experts were identified using pre-defined eligibility criteria. Priori consensus criteria were defined for each round through descriptive statistics. Following completion of round 3 a list of clinical indicators that achieved consensus were generated.

**Results:**

Thirty-eight participants were recruited across 11 countries. Thirty-five participants completed round 1 (92.1%), 32 (84.2%) round 2 and 30 (78.9%) round 3. Round 1 identified consensus (Kendall’s W coefficient of concordance 0.456; *p* < 0.001) for 10 clinical indicators out of the original 14, and 9 additional indicators were added to round 2 following content analysis of qualitative data. Round 2 identified consensus (Kendall’s W coefficient of concordance 0.749; *p* < 0.001) for 10 clinical indicators out of 19, and 1 additional indicator was added to round 3. Round 3 identified consensus for 8 indicators (Kendall’s W coefficient of concordance 0.648; *p* < 0.001). Following completion of the third round, an expert derived consensus list of 8 items was generated. Two indicators; pain variously described a burning, electric shock like and/or shooting into leg and pain in association with other neurological symptoms (e.g. pins and needles, numbness, weakness), were found to have complete agreement amongst expert participants.

**Conclusions:**

Good agreement was found for the consensus derived list of 8 clinical indicators to identify NP in low back related leg pain. This list of indicators provide some indication of the criteria upon which clinicians can identify a NP component to low back related leg pain; further research is needed for stronger recommendations to be made.

## Background

Neuropathic pain (NP) represents a substantial burden globally, to the individual and the wider economy [1]. According to the International Association for the Study of Pain (IASP), NP is defined as “pain arising from a disease or lesion of the somatosensory nervous system” [2]. Global NP prevalence reports are estimated to be between 6.9–10% [3]. Chronic low back pain is the most common type of NP disorder [4], presenting as low back related leg pain; often referred to as sciatica [5]. In comparison to low back pain alone, low back related leg pain is associated with substantially greater pain levels and poorer quality of life [5]. Furthermore, individuals with low back related leg pain with NP have been found to have a poorer prognosis compared to those with low back related leg pain without NP [5].

Low back related leg pain manifests as numerous different clinical presentations presenting as a highly heterogenous phenomenon. NP is a common pain mechanism involved in low back related leg pain, however it is not exclusive to, or a requirement of low back related leg pain [6]. The pathophysiology that governs NP in low back related leg pain is likely due to multifaceted processes including mechanical [7], ischemic [8] and neuroinflammatory [9] components. The combination of these components dictates the neurophysiological changes that occur, resulting in a presentation such as lumbar radicular pain. These patho-mechanics differ to those that would underlie a primarily nociceptive expression of low back related leg pain, such as that seen in somatic/referred pain presentations [10]. It is clear that pain mechanisms coexist in low back related leg pain [11] however in order for management to be effective, an understanding of the presence of NP is essential, as this knowledge will help to direct treatment [5]. This is highlighted by the NICE guidelines for low back pain with sciatica [12] which recommend pharmaceutical intervention to align with the NICE guidelines for NP [13], this differs to the recommendations made for low back pain alone. Furthermore, it is advocated that pain medication targeted at a pain mechanism over a specific pathology is found to be more effective [14].

Guidelines governing NP (European Federation of Neurological Societies, National Institute for Health and Care Excellence, Canadian Pain Society) uniformly recommend management that is specific to pharmaceutical intervention [15]. Therefore, accurate diagnosis of NP in low back related leg pain is essential to ensure effective management. Despite the vast interest surrounding NP there is no accepted gold standard for diagnosing NP in low back related leg pain [16]. Our recent systematic review investigated the diagnostic utility of clinical indicators to identify NP in low back related leg pain [17], finding a lack of uniformity across included studies for reference standards and terminology relating to NP in low back related leg pain. The review’s conclusion was a need for consensus regarding a reference standard to enable evaluation of diagnostic utility of clinical indicators for NP in low back related leg pain. The NP Special Interest Group (NeuSPIG) published a grading system for NP in 2008 [2] which was then revised in 2016 [18]. The grading system indicates the level of certainty (possible, probable, and definite) for which the presence of NP can be determined. A central component for all levels of certainty requires “neuroanatomically plausible” patterns of pain/sensory symptoms, however there is evidence to refute classic neuroanatomic patterns of symptom distribution for example, for entrapment neuropathies [19]. Furthermore, to achieve a ‘definite’ level of certainty the use of diagnostic tests is recommended; including the use of imaging, however it is well understood that lumbar spine imaging can highlight structural abnormalities that do not necessarily correlate with symptom presentation [20], and therefore this grading system must be used with caution.

Smart et al. [21] generated a list of fourteen clinical indicators to identify NP in musculoskeletal pain, in a Delphi survey of experts. This research was aimed at identifying NP in musculoskeletal pain and therefore these indicators may not be specific to low back related leg pain. The clinical classification accuracy of these indicators were investigated in low back pain patients with and without leg pain [22], finding that a cluster of two symptoms and one sign demonstrated a high level of classification accuracy. However, this study has been highlighted as being at risk of bias, due to clinicians not being blinded to the results of the reference standard before conducting the index test, therefore the results must be observed with caution [17]. Smart et al’s original list of clinical indicators to identify NP in musculoskeletal pain serves as a useful starting point, however an update of this list is required. Firstly, the list of clinical indicators must be specific to NP in low back related leg pain not to musculoskeletal pain. Secondly, elements of Smart et al’s list have been disputed due to evidence that has since been published, as highlighted in this study’s protocol [23]. Therefore, an updated version of Smart et al’s Delphi is needed in order to devise a list of clinical indicators specific to identifying NP in low back related leg pain. In the absence of a gold standard, an updated expert derived consensus list will help to address the gap in the literature. Expert opinion provides an effective means to address areas of clinical uncertainty through consensus [24].

### Aim

To achieve expert consensus on a list of clinical indicators to identify NP in low back related leg pain.

## Methods

### Design

**Fig. 1:**
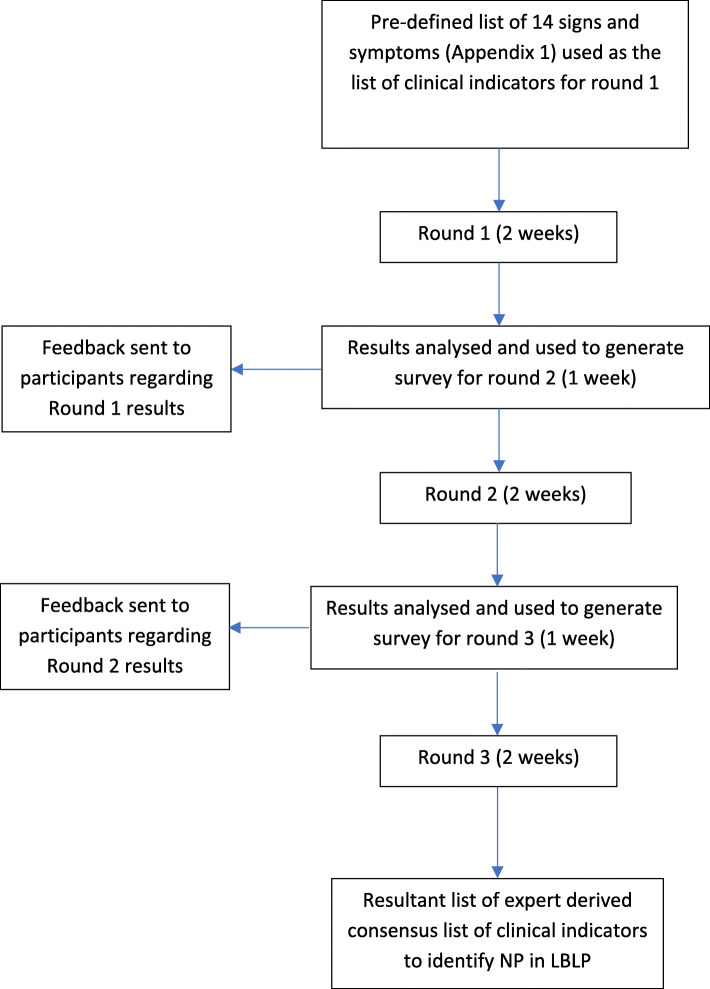
Stages of Modified Delphi study

A modified Delphi study was conducted, informed by a pre-defined and published protocol [23] and is reported in line with the Conducting and Reporting Delphi Studies (CREDES) recommendations to ensure rigour. RedCap (https://www.project-redcap.org) was used as a platform to construct and distribute three rounds of surveys to participants. Participants were given 2 weeks to complete each round and 1 week was allocated per round for data analysis. Results from rounds 1 and 2 informed the construction of the subsequent round survey. Non-responders were sent 3 reminders per round at equally distributed intervals. Agreement was defined according to pre-defined consensus criteria per round using descriptive statistics. At the end of round 3, participants ranked the patient history and clinical examination indicators in terms of importance. Clinical indicators were retained for subsequent rounds if consensus was achieved and were removed if consensus was not achieved. Feedback was given to participants for rounds 2 and 3 regarding the previous round's results. The clinical indicators generated following round 3 were collated to create the final list of clinical indicators to identify NP in low back related leg pain. Figure 1 depicts the procedure in stages of the modified Delphi study.

### Steering committee

The steering committee made collective decisions regarding methodology, data analysis and quality assurance. The steering committee was comprised of the 5 authors of this study. The lead author (JM) and co-author (TN) were students (MRes and PhD) at the University of Birmingham. The remaining 3 members of the steering committee were comprised of senior academics at the University of Birmingham (AR, DF, NH), who were also were acting in a supervisory capacity for the lead author (JM).

### Participants

In line with the CREDES recommendations, experts were defined according to pre-defined eligibility criteria. The eligibility criteria were informed by previous similar studies [25, 26], and agreed upon by the steering committee.

Eligibility criteria (≥ 1 criterion required for inclusion):

- ≥ 2 peer-reviewed publications in the field of NP in low back related leg pain [25] or
- ≥ 10 years experience working in a pain/musculoskeletal outpatient service

Expert participants were sought globally and from a variety of different professional backgrounds (physiotherapy, medicine, academia and other healthcare professional). An a priori minimum number of participants was defined at 30, as agreed by the steering committee; and based upon previous published research [27]. An upper limit for participant numbers was not defined.

### Recruitment

The recruitment period duration was 6 weeks. A snowballing strategy was adopted by the 2 recruiting authors (JM, TN), contacting experts through:

- Emailing authors of published systematic reviews relating to NP in low back related leg pain
- Posting social media expressions of interest

Experts contacted were also requested to recommend peers who satisfied the eligibility criteria. Participation was confirmed following receipt of a signed consent form, conflict of interest form and participant information form.

### Procedure

A pilot was conducted prior to commencement of the study. Eight students with musculoskeletal expertise (MRes/MSc) were invited to complete the round 1 survey over a 1-week period and asked to feedback any points to help improve the usability of the survey. Feedback was collated and no modifications were indicated.

#### Round 1

**Fig. 2:**
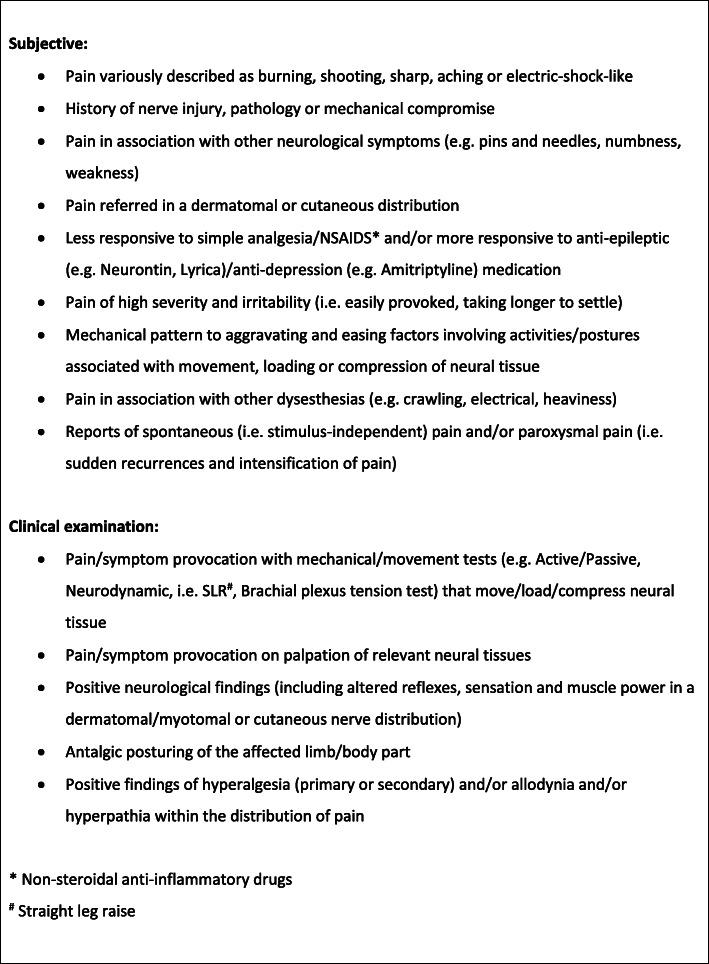
Smart’s original consensus criteria [21]

The survey for round 1 was constructed in RedCap using a list of pre-defined criteria (see Fig. 2) [21]. Smart et al’s list of clinical indicators were used as the initial indicators in Round 1 of this study. This list was utilised as it consists of subjective and objective indicators which can be easily translated into clinical practise. Furthermore, the list has been generated through a similar designed study and therefore easily compared to the current study. Specific screening tools were not used as there is no consensus regarding superiority of one over the other, and furthermore none are validated in identifying NP in low back related leg pain [17]. The 2016 NeuPSIG recommendations for diagnosing NP were not used in this study. Imaging is a requirement for certain criteria within these recommendations, and thus not clinically applicable to all [18].

Distribution of all surveys were administered through RedCap and participants were given 2 weeks to complete each survey. Participants who did not respond were removed from this study. Consensus was assessed through analysing descriptive statistics against pre-defined criteria for consensus. Content analysis was used to analyse data from the free text boxes; themes were identified which helped to inform the construction of the round 2 survey. Results of the descriptive statistics and content analysis were fed back to the steering committee and discussed before constructing the round 2 survey. Feedback regarding round 1 was sent to all participants as part of the round 2 survey.

#### Round 2

Data analysis and steering committee feedback/discussion were completed as per round 1 and the round 3 survey was constructed. Feedback regarding round 2 was sent to all participants as part of the round 3 survey, to those who completed round 2.

#### Round 3

Data analysis and steering committee feedback/discussion were completed as per rounds 1 and 2. An additional component to the round 3 survey was implemented; at the end of the survey all the indicators were divided into patient history and clinical examination sections and participants were invited to rank them in terms of their importance. On completion of round 3, and following analysis a list of indicators that achieved consensus were generated.

### Data management and analysis

All data analyses were initially completed by the lead investigator (JM) and the second reviewer (TN). Descriptive data were inputted into SPSS (Version 25.0. Armonk, NY: IBM Corp.) for statistical analysis. Qualitative data were collated in a Word document for analysis.

A priori consensus criteria were set based on Likert scale scores (5 = Strongly agree, 4 = Agree, 3 = No opinion, 2 = Disagree, 1 = Strongly disagree) for each round using descriptive statistics consisting of median, inter quartile range (IQR) and percentage of agreement, in line with previously published research (Table 1) [25]. Kendall’s W coefficient of concordance (statistical significance set at *p* < 0.05) was used for each round to assess overall agreement between participants and, was also used to assess agreement with ranking in round 3. Kendall’s W coefficient of concordance agreement figures were interpreted as poor agreement ≤ 0.20, fair agreement 0.21 to 0.40, moderate agreement 0.41 to 0.60, good agreement 0.61 to 0.80, and very good agreement 0.81 to 1.00 [28].

**Table 1:** Priori consensus criteria per round

Round 1	Median value of participants Likert scale data ≥ 3
	Percentage of agreement 50%
Round 2	Median value of participants Likert scale data ≥ 3.5
	IQR value of participants Likert scale data ≤ 2
	Percentage of agreement 60%
Round 3	Median value of participants Likert scale data ≥ 4
	IQR value of participants Likert scale data ≤ 1
	Percentage of agreement 70%

## Results

### Participants

Thirty-eight participants were recruited across 11 countries, their experience working with NP in low back related leg pain ranged from 10 to 35 years and they comprised primarily of physiotherapists and researchers/academics. Participants who were resident in the United Kingdom formed the majority of the study cohort (71%). Most participants were physiotherapists (68%), one was an osteopath and another was a neuroscientist. The remaining participants were working in academia (29%). The most common highest academic qualification amongst participants was a MSc (55%) followed by a PhD (37%). The most common time period to be working within the relevant field was 10–15 years (74%). Of the included participants, 26% had ≥ 2 peer reviewed journals published relating to NP in low back related leg pain. See Table 2 for participant characteristics.

**Table 2:** Characteristics of participants

Characteristics of expert participants	Total number
Gender	
Male	*n* = 24
Female	*n* = 14
Age	
30–39	*n* = 8
40–49	*n* = 21
50–59	*n* = 8
≥ 60	*n* = 1
Occupation	
Physiotherapist	*n* = 12
Extended scope Physiotherapist	*n* = 9
Consultant Physiotherapist	*n* = 4
Lecturer	*n* = 5
Research fellow	*n =* 1
Professor	*n* = 3
PhD student	*n* = 2
Neuroscientist	*n* = 1
Osteopath	*n* = 1
Country of origin	
UK	*n* = 20
Ireland	*n* = 2
Australia	*n* = 6
India	*n* = 2
Switzerland	*n* = 2
Norway	*n* = 1
Netherlands	*n* = 1
USA	*n* = 1
Italy	*n* = 1
South Africa	*n* = 1
Greece	*n* = 1
Highest academic qualification	
BSc	*n* = 1
PGDip	*n* = 2
MSc	*n* = 21
MRes	*n* = 1
PhD	*n* = 13
Time period working with NP	
10–15 years	*n* = 27
16–20 years	*n* = 3
> 20 years	*n* = 8
Peer review journal > 2	
0	*n* = 28
2–5	*n* = 4
6–10	*n* = 0
> 10	*n* = 6

### Round 1

Thirty-five participants completed round 1 (92.1% response rate), 3 participants did not complete the survey. Consensus was achieved with Kendall’s W coefficient of concordance 0.456 (*p* < 0.001), for 10 clinical indicators out of the original 14 (Table 3). Four indicators therefore did not achieve consensus (Table 3).

**Table 3:** Round 1 descriptive statistics

Round 1 criteria for consensus:			
✓ Median value of participants Likert scale data ≥ 3			
✓ Percentage of agreement 50% (Wiangkham et al., 2016 [25])			
Clinical indicator	Median	Percentage of agreement	Consensus achieved
Pain variously described as burning, shooting, sharp, aching or electric-shock-like	4	85.7%	Y
History of nerve injury, pathology or mechanical compromise	4	77.2%	Y
Pain in association with other neurological symptoms (e.g. pins and needles, numbness, weakness)	4	77.2%	Y
Pain referred in a dermatomal or cutaneous distribution	3	48%	N
Less responsive to simple analgesia/NSAIDS and/or more responsive to anti-epileptic (e.g. Neurontin, Lyrica)/anti-depression (e.g. Amitriptyline) medication	3	39%	N
Pain of high severity and irritability (i.e. easily provoked, taking longer to settle)	4	54.3%	Y
Mechanical pattern to aggravating and easing factors involving activities/postures associated with movements, loading or compression of neural tissue	3	42.9%	N
Pain in association with other dysesthesias (e.g. crawling, electrical, heaviness)	4	68.6%	Y
Reports of spontaneous pain (i.e. stimulus independent) and/or paroxysmal pain (i.e. sudden recurrences and intensification of pain)	4	51.4%	Y
Pain/symptom provocation with mechanical/movement tests (e.g. Active/Passive, Neurodynamic, i.e. SLR, Brachial plexus tension test) that move/load/compress neural tissue	4	65.7%	Y
Pain/symptom provocation on palpation of relevant neural tissues	4	51.4%	Y
Positive neurological signs (including altered reflexes, sensation and muscle power in dermatomal/myotomal or cutaneous nerve distribution)	4	63.8%	Y
Antalgic posturing of the affected limb/body part	2	37.1%	N
Positive findings of hyperalgesia (primary or secondary) and/or allodynia and/or hyperpathia within the distribution of pain	4	57.2%	Y

Following content analysis of round 1 data 4 themes were identified.

#### Pain descriptors need to be split into separate indicators

“............too many options in this sentence. Whilst I agree with shooting and electric, many patients describe non-neuropathic low back pain as aching, burning, sharp, and sometimes shooting” (Participant 12)

“some of these descriptors I would associated with neuropathic pain but not all of them” (Participant 10)

“ … …… … aching certainly not neuropathic, so this question is difficult to answer, for burning, shooting, electric shock, I would strongly agree” (Participant 3)

#### Latency of pain following aggravating factor

“can have a latency affect - which needs to be considered” (Participant 2)

“Latency of pain - nerve pain will often manifest the day after the aggravating activity” (Participant 26)

#### Positive small fibre testing

“one could also test the small fibres, as NDTs are not enough in order to assess probably a UNT or SLR properly” (Participant 33)

“need to continue to develop clinically feasible methods for assessing function of smaller diameter afferent fibers as part of sensory examination” (Participant 22)

#### Indicators not exclusive to NP

“Agree that these terms would be used to describe nerve root related leg pain, but not exclusively” (Participant 9)

“I think this is a multifactorial and multi symptomatic problem. It is an overall picture involving a number of points made above but not exclusively all” (Participant 23)

Following content analysis nine additional indicators were included in the round 2 survey (Table 4). At the end of each indicator “increases your index of suspicion that there is a NP component to low back related leg pain” was added.

**Table 4:** Round 2 descriptive statistics

Round 2 criteria for consensus include:				
✓ Median value of participants Likert scale data ≥ 3.5				
✓ IQR value of participants Likert scale data ≤ 2				
✓ Percentage of agreement 60% (Wiangkham et al., 2016 [25])				
Clinical indicator	Median	IQR	Percentage of agreement	Conesus achieved
**Pain described as burning**	4	1	90.9%	Y
**Pain described as shooting**	4	1	73.8%	Y
**Pain described as sharp**	3	1	18.2%	N
**Pain described as aching**	2	2	12.1%	N
**Pain described as electric-shock-like/electrical**	4	1	93.9%	Y
**Pain described as cramping**	3	2	30.3%	N
**Pain described as crawling**	4	1	66.7%	Y
**Pain described as heaviness**	3	2	33.3%	N
History of nerve injury, pathology or mechanical compromise	5	1	90.9%	Y
Pain in association with other neurological symptoms (e.g. pins and needles, numbness, weakness)	5	1	96.9%	Y
Pain of high severity and irritability (i.e. easily provoked, taking longer to settle)	4	1	72.8%	Y
Reports of spontaneous pain (i.e. stimulus independent) and/or paroxysmal pain (i.e. sudden recurrences and intensification of pain)	4	2	72.7%	Y
Pain/symptom provocation with mechanical/movement tests (e.g. Active/Passive, Neurodynamic, i.e. SLR, Brachial plexus tension test, prone knee bend)	4	1	67.9%	Y
Pain/symptom provocation on palpation of relevant neural tissues	4	1	57.6%	N
Positive neurological signs (including altered reflexes, sensation and muscle power in dermatomal/myotomal or cutaneous nerve distribution)	5	1	94%	Y
Positive findings of hyperalgesia (primary or secondary)	3	2	42.4%	N
Allodynia and/or hyperpathia within the distribution of pain	4	2	66.7%	Y
**Latent pain response to aggravating factor**	4	1	51.5%	N
**Positive small fibre nerve testing findings (Hot/cold etc....)**	4	1	84.8%	Y

*SLR* Straight leg raise. Additional indicators indicated in bold

### Round 2

Thirty-two participants completed round 2 (84.2% response rate), 3 participants did not complete the survey. Consensus was achieved with Kendall’s W coefficient of concordance 0.749 (*p* < 0.001) for 12 clinical indicators out of 19 (Table 4). Seven indicators did not achieve consensus (Table 4).

Following content analysis of round 2 data, 2 themes were identified.

#### History of nerve lesion or disease of somatosensory nervous system is difficult to identify/define

“this is of course the IASP definition of NP, that it has to be associated with a lesion or disease of SSNS, tricky bit is that often in clinics it is not so straightforward to demonstrate the lesion/disease” (Participant 29)

“mechanical compromise is difficult to define and will be interpreted differently by various professions / therapists” (Participant 10)

“I'm not sure exactly what mechanical compromise means here” (Participant 31)

#### As well as a history of nerve injury, pathology or mechanical compromise other factors can contribute to the development of NP in low back related leg pain

“metabolic changes / diabetic or autoimmune diseases” (Participant 8)

“recent or past chemotherapeutic drugs prescription” (Participant 11)

“immune compromise e.g. HIV” (Participant 19)

Following content analysis one additional indicator was included for round 3 (Table 5).

**Table 5:** Round 3 descriptive statistics

Round 3 criteria for consensus include:						
✓ Median value of participants Likert scale data ≥ 4						
✓ IQR value of participants Likert scale data ≤ 1						
✓ Percentage of agreement 70% (Wiangkham et al., 2016 [25])						
Clinical indicator	Median	IQR	Percentage of agreement (%)	Consensus achieved	Ranking patient history indicators	Ranking clinical examination indicators
Pain variously described as burning, electric shock like and/or shooting into leg	5	1	100	Yes	2	
Pain described as crawling or another unpleasant abnormal sensation (as a common example of dysesthesia)	4	1	90.3	Yes	4	
History of nerve injury, pathology or mechanical compromise at the region of the nerve root/or other nervous tissue around the lumbar spine that can refer into the leg	5	1	96.7%	Yes	3	
**In a patient with low back related leg pain does the pre-existing knowledge of metabolic (e.g. diabetes, vitamin deficiencies), hormonal (e.g. thyroid), genetic (e.g. channelopathies), pharmacological (antimetabolities), chemical (e.g. chemotherapy) conditions**	3	1	48.5%	No	7	
Pain in association with other neurological symptoms (e.g. pins and needles, numbness, weakness)	5	1	100%	Yes	1	
Pain of high severity and irritability (i.e. easily provoked, taking longer to settle)	4	2	64.5%	No	6	
Reports of spontaneous pain (i.e. stimulus independent) and/or paroxysmal pain (i.e. sudden recurrences and intensification of pain)	4	1	71.1%	Yes	5	
Pain/symptom provocation with mechanical/movement tests (e.g. Active/Passive, Neurodynamic, i.e. SLR, Brachial plexus tension test)	4	1	67.8%	No		3
Positive neurological signs (including altered reflexes, sensation and muscle power in dermatomal/myotomal or cutaneous nerve distribution)	5	1	90.4%	Yes		1
Allodynia and/or hyperpathia within the distribution of pain	4	1	74.2%	Yes		4
A loss of function of small fibre testing	4	1	77.4%	Yes		2

Furthermore, the pain descriptors that achieved consensus were combined into one indicator.

Also, the indicator “History of nerve injury, pathology or mechanical compromise increases your index of suspicion that there is a NP component to low back related leg pain” was modified to further specify the location of compromise.

### Round 3

Thirty participants completed round 3 (78.9% response rate), two participants did not complete the survey. Consensus was achieved for 8 indicators, Kendall’s W coefficient of concordance 0.648 (*p* < 0.001) (Table 5). Three indicators did not achieve consensus (Table 5).

Following content analysis 3 themes were identified.

#### Other factors such as metabolic, hormonal etc. … contributors are too general and not specific enough when identifying NP in low back related leg pain

“Will be used in the clinical reasoning process, but need other descriptors or indicators. In isolation, not indicative of neuropathic pain” (Participant 3)

“Although diabetes and radiotherapy can result in NP, this has is not low back-related”, “I suppose it may make you consider that their nervous system is more vulnerable, but whether it means that they have a NP component to their low back pain is unclear” (Participant 11)

#### High severity and irritability not specific enough to identify NP in low back related leg pain

“it can be part of the picture of neuropathic pain, but not totally discriminatory” (Participant 6)

“ … …… … these components are too non-specific to even increase my index of suspicion of a neuropathic component to low back-related leg pain. While nerve-related problems can often be higher on the severity and irritability scales, non-neural/nociceptive problems can also be high on these scales” (Participant 28)

#### Neurodynamic testing

“I would not consider a positive lower limb neurodynamic tests to be relevant here. As previously mentioned, a negative SLR/Slump tests etc. does not mean that NP is not present esp. In the presence of nerve function loss, and its presence does not tell you for sure that there is NP component as we know many people with positive. Neurodynamic tests do not have NP. So whilst I agree that its presence may raise my suspicion, it is other factors as well that would confirm its presence” (Participant 14)

“ …………heightened nerve mechanosensitivity is NOT the same as neuropathic pain. Quite often heightened nerve mechanosensitivity is associated with nociceptive pain. This is a huge fallacy in contemporary Physiotherapy, and often misinterpreted...see many studies which show that neurodynamic tests can be negative in patients with properly diagnosed nerve lesions (so by your (IASP) definition have neuropathic pain...)” (Participant 15)

Following completion of the third round, an expert derived consensus list of 8 items was generated. See Table 6 for the list of clinical indicators.

**Table 6:** List of expert derived indicators to identify NP in low back related leg pain

**Pain variously described as burning, electric shock like and/or shooting into leg** (percentage of agreement 100%)	
**Pain described as crawling or another unpleasant abnormal sensation (as a common example of dysesthesia)** (percentage of agreement 90.3%)	
**History of nerve injury, pathology or mechanical compromise at the region of the nerve root/or other nervous tissue around the lumbar spine that can refer into the leg** (percentage of agreement 96.7%)	
**Pain in association with other neurological symptoms (e.g. pins and needles, numbness and weakness)** (percentage of agreement 100%)	
**Reports of spontaneous pain (i.e. stimulus independent) and/or paroxysmal pain (i.e. sudden recurrences and intensification of pain)** (percentage of agreement 71%)	
**Positive neurological signs (including altered reflexes, sensation and muscle power in dermatomal/myotomal or cutaneous nerve distribution)** (percentage of agreement 90.4%)	
**Allodynia and/or hyperpathia within the distribution of pain** (percentage of agreement 74.2%)	
**Loss of function of small fibre nerve testing** (percentage of agreement 77.4%)	

### Consensus on ranking

Rankings of clinical indicators were split into patient history (Table 7) and clinical examination indicators (Table 8). The two patient history indicators ranked as the most important were “Pain in association with other neurological symptoms (e.g. pins and needles, numbness, weakness)” and “Pain variously described as burning, electric shock like and/or shooting into leg increases your index of suspicion of a NP component to low back related leg pain.” The clinical examination indicator ranked as the most important was “Positive neurological signs (including altered reflexes, sensation and muscle power in dermatomal/myotomal or cutaneous nerve distribution).” Ranking order correlated with percentage of agreement for all clinical indicators.

**Table 7:** Ranking of patient history examination clinical indicators that achieved consensus

1) Pain in association with other neurological symptoms (e.g. pins and needles, numbness, weakness) increases your index of suspicion that there is a NP component to low back related leg pain	
2) Pain variously described as burning, electric shock like and/or shooting into leg increases your index of suspicion of a NP component to low back related leg pain.	
3) History of nerve injury, pathology or mechanical compromise at the region of the nerve root/or other nervous tissue around the lumbar spine that can refer into the leg increases your index of suspicion of a NP component to low back related leg pain.	
4) Pain described as crawling or another unpleasant abnormal sensation (as a common example of dysesthesia) increases your index of suspicion of a NP component to low back related leg pain.	
5) Reports of spontaneous pain (i.e. stimulus independent) and/or paroxysmal pain (i.e. sudden recurrences and intensification of pain) increases your index of suspicion that there is a NP component to low back related leg pain	

Ranking of importance: 1 = highest important, 5 = least important

**Table 8:** Ranking for clinical examination clinical indicators that achieved consensus

1) Positive neurological signs (including altered reflexes, sensation and muscle power in dermatomal/myotomal or cutaneous nerve distribution) increases your index of suspicion that there is a NP component to low back related leg pain	
2) A loss of function of small fibre testing increases your index of suspicion that there is a NP component to low back related leg pain	
3) Allodynia and/or hyperpathia within the distribution of pain increases your index of suspicion that there is a NP component to low back related leg pain	

Ranking of importance: 1 = highest important, 3 = least important

Agreement was assessed using Kendall’s W coefficient of concordance. For patient history indicators alone, agreement was 0.390 (fair agreement). For clinical examination indicators alone agreement was 0.204 (fair agreement). Combining both patient history and clinical examination indicators ranking results together agreement was 0.431 (moderate agreement).

## Discussion

This is the first study to derive an expert consensus list of clinical indicators of possible NP specifically in patients with low back related leg pain. Over the 3 rounds the overall response rate was 78.9% which is above the recommended acceptable response rate for Delphi studies [29]. The outcome of this study is an expert derived list of clinical indicators to identify possible NP in low back related leg pain, with pragmatic implications for both clinical practice and contemporary research once further research is conducted to support this list of clinical indicators.

The findings of this study identify complete agreement (100%) for the use of pain descriptors and high agreement (90.3%) for the use of dysesthesia descriptors for identifying NP in low back related leg pain. The use of pain descriptors and descriptors of dysesthesia are commonly used in NP screening tools; such as the Leeds Assessment of Neuropathic Symptoms and Signs (LANSS) [30], PainDETECT [31] and the StEP tool [32]. Despite being a key feature of many screening tools, evidence supporting the use of pain/dysesthesia descriptors in identifying NP in low back related leg pain is limited [17]. There is moderate level evidence to support the use of the StEP tool, which encompasses the aforementioned descriptors, in diagnosing lumbar radicular pain, demonstrating high sensitivity (92%) and specificity (97%) values [17]. However, the StEP tool also includes numerous other patient history and clinical examination components and thus the diagnostic accuracy of the descriptors alone remain unclear. A difficulty associated with use of specific pain/dysesthesia descriptors lies in linguistic/cultural differences, which was highlighted in the qualitative data from participants in this study. When interpreting pain descriptors, in different languages or in the same language but in a different country, the descriptors may not always depict the culture’s orientation and consequently the meaning may become contaminated [33]. High levels of agreement in this study support the use of pain descriptors and descriptors of dysesthesia in identifying NP in low back related leg pain. Further research is needed in order to assess the diagnostic utility of these descriptors to enable stronger recommendations to be made regarding their use.

History of nerve injury, pathology or mechanical compromise was a clinical indicator which remained from Smart et al’s [21] original list, demonstrating high agreement (96.7%). This indicator has been demonstrated to have high sensitivity (86.3%) and specificity (96%) diagnosing peripheral NP in low back pain, with or without leg pain, when used as part of a cluster of 3 clinical indicators [22]. However, a low level of evidence supports this cluster of clinical indicators when assessed using the Grading of Recommendations Assessment, Development and Evaluation (GRADE) [17]. A degree of ambiguity, highlighted by participants in this study, surrounds the extent to which the nerve needs to be compromised to be neuropathic and how this is identified in the absence of obvious trauma. Heightened nerve mechanosensitivity is considered a primarily nociceptive driven phenomenon, in which nociceptors in the nervi nevorum are activated as a consequence of the nerve itself being compromised [19]. Despite being considered primarily nociceptive, heightened nerve mechanosensitivity could be interpreted as satisfying the indicator provided above. Through the Delphi study this indicator was modified to specify the region and structures involved. A high level of agreement amongst participants supports the use of this indicator. Research to investigate the diagnostic utility of this indicator in isolation is needed as well as clarity regarding what constitutes nerve compromise.

Pain in association with other neurological symptoms (e.g. pins and needles, numbness, weakness) and positive neurological signs (including altered reflexes, sensation and muscle power in dermatomal/myotomal or cutaneous nerve distribution) both demonstrated high agreement (100 and 90.4% respectively) and were ranked as the most important patient history and clinical examination indicators respectively (moderate agreement Kendalls coefficient 0.431). Importantly these indicators were highlighted to increase the index of suspicion of NP in low back related leg pain but it was widely acknowledged amongst participants that positive neurological findings can occur without NP and vice versa. Evidence surrounding positive neurological symptoms/findings in NP pertains largely to sensory findings [34], with reflex and motor loss suggestive of radiculopathy, which does not necessitate NP [35]. This is further supported by two systematic reviews investigating subjective and objective indicators to identify peripheral NP [22] and lumbosacral nerve root compression [36] respectively, clusters of signs and symptoms were generated in which motor/reflex loss were not featured but a common finding in both studies was sensory symptoms in a dermatomal distribution. Interestingly in this study a clinical indicator removed after round 1 was “Pain referred in a dermatomal or cutaneous distribution.” Our recent systematic review found that literature surrounding the diagnostic utility of neurological signs and symptoms in identifying NP in low back related leg pain is sparse, with studies investigating these indicators reporting them in clusters, not in isolation [17]. The high level of agreement amongst participants, as well as being ranked the highest patient history and clinical examination indicators, supports the use of both these indicators in identifying NP in low back related leg pain. However further research is needed to investigate the diagnostic utility of neurological symptoms and signs, and which of these symptoms and signs are relevant in identifying NP in low back related leg pain.

Loss of function of small fibres achieved consensus in this study, demonstrating moderate levels of agreement (77.4%). Through the Delphi process, this was distinct from the ‘positive neurological signs’ clinical indicator, as feedback identified that testing was not routine in clinical practice; however theoretically this sits within a neurological examination. It is well established that small fibre degeneration occurs before large fibres in entrapment neuropathies [19]. Furthermore, small fibre nerve testing correlates more accurately with the IASP’s definition for NP as it tests exclusively sensory fibres. Small fibre sensory testing is commonly used in experimental research studies investigating NP through means such as quantitative sensory testing (QST) [37] and skin biopsy [38]. However as this list of indicators is directed not only at researchers but a clinical population as well, the use of a bedside clinical examination remains the most relevant. Zhu et al. [39] found significant correlations between clinical sensory tests and QST when evaluating somatosensory dysfunction in 3 cohorts of patients (carpal tunnel syndrome, lumbar radicular and non-specific arm and neck pain). However, not all components of the clinical sensory tests were found to be useful, with variations in findings between patient cohorts highlighting the need to consider condition specific parameters. Standardised qualitative sensory testing (SQST) was found to have low/moderate sensitivity (62%) and high specificity (95%) when detecting lumbar lateral stenosis of the L5 nerve root. However due to indirectness of the evidence, a low level of evidence supports the use of SQST in diagnosing lumbar lateral stenosis of the L5 nerve root [17]. This Delphi supports the use of this clinical indicator. However further research into what constitutes an effective battery of clinical tests of the small fibres in identifying NP in low back related leg pain is needed and importantly what the diagnostic utility of these tests are.

Reports of spontaneous pain (i.e. stimulus independent) and/or paroxysmal pain (i.e. sudden recurrences and intensification of pain) just achieved consensus (71%). This indicator is supported by its use in numerous NP screening tools; Neuropathic Pain Questionnaire (NPQ) [40], LANSS [31], StEP [33] and painDETECT [32]. Furthermore, the underlying physiology of NP is commonly associated with ectopic firing of the primary afferent nerve following peripheral nerve insult. Sites of ectopic firing develop in response to nerve injury, occurring at multiple sites including; injury site, dorsal root ganglia [41] and in the surrounding non-injured afferents [42]. Compelling evidence to support spontaneous pain in clinical presentations of NP in low back related leg pain is however minimal. Moderate level evidence supports the use of a cluster of 8 subjective signs, of which paroxysmal pain is one, when diagnosing lumbosacral nerve root compression, demonstrating moderate/high sensitivity (72%) and specificity (80%) values. However, the diagnostic utility of this indicator is yet to be investigated in isolation [17], and this lack of evidence was also highlighted by participants in this study. This Delphi study supports the use of this indicator to increase the index of suspicion of NP in low back related leg pain however agreement was only just achieved. Further research is needed to investigate the diagnostic utility of this indicator in order for a stronger recommendation to be made.

The indicator of allodynia and hyperpathia within the distribution of pain just achieved consensus (74.2%). Attal et al. [43] found in a study involving participants with NP disorders, including lumbar radiculopathy, 55% were found to have brush evoked allodynia. Jensen and Finnerup [44] suggested that in NP disorders areas of allodynia and hyperpathia provide a measure of those structures within the nervous system where signs of neuronal hyperexcitability are present. A common theme identified widely amongst participants was that the indicator is also conducive with nociplastic pain/central sensitisation and that evidence surrounding the presence of allodynia and hyperpathia in identifying NP in low back related leg pain is limited. These findings are supported by our systematic review in which allodynia and hyperpathia are not featured as an index test in any of the included studies investigating NP in low back related leg pain [17]. Allodynia and hyperalgesia/hyperpathia are not limited to NP and therefore the use of this indicator must be considered with other clinical signs and as a means to increase index of suspicion of NP in low back related leg pain. This review supports the use of this indicator in identifying NP in low back related leg pain, however as agreement was only marginally achieved this should reflect the index of suspicion when used.

### Study findings compared to Smart et al’s (2010) original list

From Smart et al’s original list 7 indicators were retained, 1 additional indicator was added and 7 were removed. Of the 7 indicators retained, 3 indicators remained unchanged from the original list; ‘Pain in association with other neurological symptoms (e.g. pins and needles, numbness, weakness),’ ‘Reports of spontaneous pain (i.e. stimulus independent) and/or paroxysmal pain (i.e. sudden recurrences and intensification of pain)’ and ‘Positive neurological signs (including altered reflexes, sensation and muscle power in dermatomal/myotomal or cutaneous nerve distribution).’ Two of the indicators were modified slightly to specify a low back related leg pain component to the indicator; ‘Pain variously described as burning, electric shock like and/or shooting into leg’ and ‘History of nerve injury, pathology or mechanical compromise at the region of the nerve root/or other nervous tissue around the lumbar spine that can refer into the leg.’ Two indicators were worded slightly differently to improve readability/comprehension following participant feedback; ‘Pain described as crawling or another unpleasant abnormal sensation (as a common example of dysesthesia)’ and ‘Allodynia and/or hyperpathia within the distribution of pain.’ One indicator was added to this list which was not on Smart et al’s original list; ‘Loss of function of small fibre nerve testing.’ The evidence surrounding the above indicators have been described earlier in the discussion.

Seven indicators were removed from Smart et al’s original list; ‘pain referred in a dermatomal or cutaneous distribution,’ ‘Less responsive to simple analgesia/NSAIDS and/or more responsive to anti-epileptic (e.g. Neurontin, Lyrica)/anti-depression (e.g. Amitriptyline) medication,’ ‘Pain of high severity and irritability (i.e. easily provoked, taking longer to settle),’ ‘Mechanical pattern to aggravating and easing factors involving activities/postures associated with movement, loading or compression of neural tissue,’ ‘Pain/symptom provocation with mechanical/movement tests (e.g. Active/Passive, Neurodynamic, i.e. SLR, Brachial plexus tension test) that move/load/compress neural tissue,’ ‘Pain/symptom provocation on palpation of relevant neural tissues’ and ‘Antalgic posturing of the affected limb/body part.’ The differences between Smart et al’s list and the list generated from the current Delphi may be reflective of the difference in phenomena of interest in each study (NP in low back related leg pain and NP in musculoskeletal pain). Furthermore, there is a nine-year time difference between the two studies and therefore the resultant lists may be reflective of the evidence available at that particular time. Finally, the differences between lists could be attributed to the expert populations in each study, which were both defined differently.

In addition to the reasons provided for variation between each list, specific evidence pertaining to each indicator has been highlighted as possible reasons for why the indicator was removed from the original list. Pain referred in a dermatomal or cutaneous distribution has been disputed as an indicator for NP with evidence in both animal and human studies describing extraterritorial spread of symptoms in response to entrapment neuropathies [19]. Evidence surrounding neurodynamic testing and nerve palpation in relation to identifying NP is sparse however it is well established that these tests have low diagnostic validity and thus may not be a useful clinical indicator [19]. The removal of the indicator regarding pharmacotherapy may be consistent with the lack of evidence supporting the efficacy of any particular medication in NP, highlighted in a systematic review by Finnerup et al. [17]. The remaining indicators surrounding antalgic postures, mechanical aggravating factors and high irritability are not described in any of the contemporary screening tools or classifications pertaining to NP and have not been investigated in isolation for their diagnostic accuracy which may be the reason for their removal from the list [14].

### Strengths and limitations

Strengths of this study include the use of a robust methodology which adhered to a pre-defined published protocol [23]. The study is reported in line with the CREDES recommendations, which is the only reporting guidance tool available for Delphi studies [45]. Finally, on completion of this study a list of clinical indicators was generated (good agreement) and, alongside the findings of our recent systematic review, recommendations to inform future research have been made.

A limitation to this study was that the expert participants were largely made up of physiotherapists and therefore not fully representative of all healthcare professionals. The study recruited national and international experts with representatives from research/academia and clinical practice, however a large proportion of the expert participants were from the UK and therefore consensus may not be fully representative of internationally accepted opinion. A further limitation to this study was the size of the participant sample. When compared to other Delphi studies related to low back pain the numbers in this study were significantly lower [46, 47].

### Clinical and research recommendations

A list of 8 clinical indicators has been agreed to identify possible NP in low back related leg pain. Higher levels of agreement were demonstrated for 5 of the clinical indicators and therefore it is recommended that the presence of these indicators should heighten the clinician’s index of suspicion compared to the other 3 indicators:

- Pain variously described a burning, electric shock like and/or shooting into leg
- Pain in association with other neurological symptoms (e.g. pins and needles, numbness, weakness)
- Pain described as crawling or another unpleasant abnormal sensation (as a common example of dysesthesia)
- History of nerve injury, pathology or mechanical compromise at the region of the nerve root/or other nervous tissue around the lumbar spine that can refer into the leg
- Positive neurological signs (including altered reflexes, sensation and muscle power in dermatomal/myotomal or cutaneous nerve distribution)

Evidence to support the use of these clinical indicators demonstrates some promise, particularly when considering pain descriptors and ‘history of nerve injury, pathology or mechanical compromise.’ However, the diagnostic utility of these indicators individually when identifying NP in low back related leg pain remains unclear [17]. Therefore, further research should evaluate the diagnostic accuracy of each of the 8 clinical indicators generated from this study, in a high quality, low risk of bias cross-sectional study. Furthermore, clarity is needed regarding aspects of the definition of NP in relation to what constitutes a lesion/disease of the somatosensory nervous system and how can this be identified. Importantly, research needs to establish the prevalence of the 8 criteria among formally diagnosed patients to give some indication of how often the criteria might be useful in practice.

This list of clinical indicators is the first of its kind, as it is the only list of indicators which are specific to identifying NP in low back related leg pain. The authors do not recommend that this list provides an alternative to other sources of evidence specific to NP (screening tools, classifications) but its use may heighten the suspicion of the presence of NP in low back related leg pain. Further research is needed before implementation into clinical practise in relation to measurement properties, such as reliability, validity, feasibility and acceptability.

The authors of this study support the use of this list of clinical indicators as a means to increase the index of suspicion for the presence of NP in low back related leg pain, however this should be interpreted alongside clinical examination findings and clinician experience. Stronger recommendations regarding this list can be made once the diagnostic utility of these indicators is investigated.

## Conclusion

Good agreement was found for the consensus derived list of 8 clinical indicators to identify possible NP in low back related leg pain. Five of the 8 clinical indicators demonstrated higher levels of agreement amongst experts and are therefore recommended to heighten the clinician’s index of suspicion relative to the other 3 indicators. Evidence to support the diagnostic utility of each of the 8 clinical indicators is limited and therefore a need for further research in this area is required for stronger recommendations to be made.

## Data Availability

No patient data sets used in this review.

## Abbreviations

NP: Neuropathic pain
IASP: International Association for the Study of Pain
NICE: The National Institute for Health and Care Excellence
NeuSPIG: Neuropathic Pain Special Interest Group
CREDES: Conducting and Reporting Delphi Studies
IQR: Inter Quartile Range
LANSS: Leeds Assessment of Neuropathic Symptoms and Signs
GRADE: Grading of Recommendations Assessment, Development and Evaluation
QST: Quantitative Sensory Testing
SQST: Standardised qualitative sensory testing
NPQ: Neuropathic Pain Questionnaire
SLR: Straight Leg Raise
